# High-Throughput NMR Assessment of the Tertiary Structure of Food Allergens

**DOI:** 10.1371/journal.pone.0039785

**Published:** 2012-07-02

**Authors:** Stefano Alessandri, Ana Sancho, Stefan Vieths, Clare E. N. Mills, Jean-Michel Wal, Peter R. Shewry, Neil Rigby, Karin Hoffmann-Sommergruber

**Affiliations:** 1 CERM, Centro di Ricerca di Risonanze Magnetiche and Department of Agricultural Biotechnology, University of Florence, Florence, Italy; 2 Institute of Food Research, Norwich, United Kingdom; 3 Paul-Ehrlich-Institut, Langen, Germany; 4 INRA, UR496 Immuno-Allergie Alimentaire, CEA/iBiTeC-S/SPI, CEA de Saclay, Gif sur Yvette, France; 5 Rothamsted Research, Harpenden, United Kingdom; 6 Department of Pathophysiology and Allergy Research, Medical University of Vienna, Vienna, Austria; University of South Florida College of Medicine, United States of America

## Abstract

**Background:**

*In vitro* component-resolved diagnosis of food allergy requires purified allergens that have to meet high standards of quality. These include the authentication of their conformation, which is relevant for the recognition by specific IgE antibodies from allergic patients. Therefore, highly sensitive and reliable screening methods for the analysis of proteins/allergens are required to assess their structural integrity. In the present study one-dimensional 1H Nuclear Magnetic Resonance (1D 1H-NMR) analysis was adopted for the assessment of overall structural and dynamic properties and authentication of a set of relevant food allergens, including non-specific lipid transfer proteins from apple, peach and hazelnut, 7/8S seed storage globulins from hazelnut and peanut, 11S seed storage globulins from hazelnut and peanut, caseins from cows' and goats' milk and tropomyosin from shrimp.

**Methodology/Principal Findings:**

Two sets of 1D 1H-NMR experiments, using 700 MHz and 600 MHz instruments at 298 K were carried out to determine the presence and the extent of tertiary structure. Structural similarity among members of the individual allergen families was also assessed and changes under thermal stress investigated. The nuclear magnetic resonance (NMR) results were compared with structural information available either from the literature, Protein Data Bank entries, or derived from molecular models.

**Conclusions/Significance:**

1D ^1^H-NMR analysis of food allergens allowed their classification into molecules with rigid, extended and ordered tertiary structures, molecules without a rigid tertiary structure and molecules which displayed both features. Differences in thermal stability were also detected. In summary, 1D ^1^H-NMR gives insights into molecular fold of proteins and offers an independent method for assessing structural properties of proteins.

## Introduction

IgE-mediated food allergies are caused by non-toxic dietary proteins of animal or plant origin. Once sensitized, predisposed individuals develop allergic symptoms upon intake. Clinical manifestations range from mild, local reactions to generalised severe, even life-threatening conditions. However, only a restricted number of proteins are capable of inducing allergic sensitization and elicitation [1].

Conventional *in vitro* allergy diagnosis is based on the detection of specific IgE antibodies directed against allergens present in total extracts of food materials. In the recent past the concept of component-resolved diagnosis, in which total extracts are replaced by panels of single allergens for each food, has been introduced aiming at improved sensitivity, reproducibility and specificity of the assay [2]–[4]. However, the quality of the test performance depends on the quality of the analytical tools used. Therefore, the concept of an allergen library was developed within the EuroPrevall project [5] which included purified members of the allergen families known as non-specific lipid transfer proteins (nsLTPs), 7S seed storage globulins, 11S seed storage globulins, caseins from cows' and goats' milk and tropomyosin from shrimp. State of the art methods for the purification of allergenic food proteins were collated and a catalogue of quality criteria was agreed, including the evaluation of analytical methods for their authentication [6].

IgE antibodies produced by allergic individuals to a food allergen display different specificities and may be directed against linear and conformational epitopes. Particularly for the conformational, discontinuous lgE epitopes a 3-dimensional structure of the purified allergen closely resembling that of the protein in its natural environment is mandatory for reliable *in vitro* diagnosis. Therefore, highly efficient methods to assess the structural integrity of these proteins are required. It is particularly important to confirm that individual batches of purified proteins resemble the naturally occurring protein in their structures and properties, displaying either rigid, intermediate or flexible tertiary structures the latter being important for allergens that may exist in partially-unfolded states induced by thermal processing [7], [8].

Nuclear Magnetic Resonance (NMR) spectroscopy is one of the main techniques used to study the structural properties of native and processed proteins and is capable of atomic resolution in aqueous solutions. i.e. under conditions similar to the physiological state. It provides information on the positions, bonds and movements of specific atoms with nuclei which have a spins different from zero, primarily ^1^H, and on their environment. Such information can be used for the characterization of a protein in terms of structural, chemical and dynamic properties [7], [8]. However, 3D-structure determination may require isotopically-labelled protein to allow such highly resolved structures to be determined. In addition, the instrumental and human resources required for this type of NMR experiment, and the concentrations of proteins that are required, also make it impossible to currently define them as “high-throughput”. An alternative is to use 1D NMR spectra as an initial screening tool, to differentiate between protein chains with rigid and ordered tertiary structures, from those that are randomly coiled and those proteins that are more mobile, disordered or flexible. 1D ^1^H-NMR spectra already contain enough information to evaluate whether a protein has some tertiary structure, although the signals are not assigned and the determination of 1D ^1^H-NMR spectra is much less time consuming than other kind of NMR experiment. Close inspection of one-dimensional ^1^H spectra also provides information about the extent of tertiary structure in partially-structured proteins, about mixtures where only one fraction is structured or about mixture of conformers like molten globules [9]. Aggregation can be also detected by observing the line width of the signals because the width of the signals in a solution NMR spectrum is correlated with the size of the molecule. Such NMR screening is considered a useful tool for identifying targets which have a high probability of producing crystals of sufficient quality for x-ray diffraction studies or are amenable to structural analysis by NMR. 1D ^1^H-NMR screening allows structural genomics and proteomics studies to be focused on the most promising targets and, therefore, significantly reduces the cost of production of novel 3D protein structures [10], [11].

Several examples of the range of structural types that are represented by allergens were chosen, from totally unstructured molecules (caseins), to proteins belonging to families characterized by a rigid and ordered tertiary structure (non specific lipid transfer proteins, nsLTP), representing the most important plant food allergens families [12], [13]. These included the nsLTPs as representatives of the prolamin superfamily and are identified as allergens in fruits, vegetables and nuts. In Rosaceae fruits they are major allergens accounting for severe allergic reactions in patients. Non specific lipid transfer proteins are stabilised by four disulfide bridges and typically resistant to thermal and enzymatic treatment [14]. The structure of Pru p 3, the major allergen from peach, was determined by X-ray crystallography [15] (PDB entries 2alg and 2b5s). Another dominant plant food allergen family are the cupins, to which seed storage proteins belong. These can be divided into two groups based on their sedimentation coefficients. These include the 7S vicilin-type globulins which are typically trimeric proteins [16] and the 11S legumin-type globulins, after post-translational processing, typically consist of six subunit pairs that interact noncovalently. Allergens from this protein family are identified from legumes, nuts and seeds. The 11S or 7S globulins can form both trimeric and hexameric structures: reversible aggregation into hexamers is possible, for 7S globulins, depending on the ionic strength and 11S globulins are assembled via a trimeric intermediate [16].

Representatives of the animal food allergen families were tropomyosins and caseins. Tropomyosins are known as major allergens in seafood and minor inhalant allergens in cockroaches and mites. Typically non vertebrate tropomyosin has a molecular mass of 33 kDa and is a homodimer of two parallel alpha helical molecules with a flexible coiled-coil structure each [17] that allows the tropomyosin to assemble around the actin filaments [18].

Caseins are typically unfolded proteins and account for the majority in the protein fraction of mammalian milk. Cow's milk casein is known as a major food allergen especially in young children. Whole caseins are mixtures of four different proteins: alpha-S1-casein (199 AA, 23.6 kDa), alpha-S2-casein (207 AA 25.2 kDa), beta-casein (209 AA, 24 kDa), k-casein (169 AA, 19 kDa; [19]). Bovine caseins consist of flexible disordered and mobile loops, with only few residues included in short portions that could be organised at the secondary level.

The aim of the current study was to apply 1D ^1^H-NMR spectroscopy to structural analysis of a range of allergens from major allergen families and to assess its potential as an effective high throughput method. The structural characteristics of the allergens within a protein family and reversible and irreversible structural transitions due to temperature changes were also investigated.

## Materials and Methods

### Preparation of the allergens

Non-specific lipid transfer proteins were purified from apple (*Malus domestica*, Mal d 3), peach (*Prunus persica*, Pru p 3) and hazelnut (*Corylus avellana*, Cor a 8) as described previously [20]–[22]. 7S and 11S seed storage globulins were purified from hazelnut (*Corylus avellana*, Cor a 11, Cor a 9) and peanut (*Arachis hypogaea*, Ara h 1, Ara h 3) as described previously [22], [23]. Caseins were purified from cows' (*Bos domesticus*, Bos d 8) and goats' milk (Whole caprine casein, WCC [24]). Tropomyosin, the major allergen from brown shrimp (*Penaeus aztecus*, Pen a 1) was produced as a recombinant protein and purified accordingly [17]. The natural and recombinant allergens were tested for their IgE-binding activity using allergic patients sera as previously described and their primary and secondary structures verified by N-terminal sequencing, MALDI-TOF-MS analysis and far-UV CD-spectroscopy [5]. An overview of the allergens used in this study, their acronyms and physicochemical parameters together with the experimental conditions of the NMR analyses are summarised in Table 1.

**Table 1:** 

Protein family	Function	Allergen* designation	Source	Mol Mass	PDB ID**	1-D-^1^H-NMR Analyses	
						Experimental conditions (protein conc.; buffer)	Structure
**Plant Food Allergens**							
Non specific lipid transfer proteins	Plant defence, transfer of lipids	Mal d 3	Apple*Malus domestica*	9 kDa	2alg (Peach)	0.08 mM; 20 mM phosphate; pH 7.4	folded
		Pru p 3	Peach*Prunus persica*	9 kDa	2alg, 2b5s	0.39 mM; 25 mM phosphate; pH 7.0	folded
		Cor a 8	Hazelnut*Corylus avellana*	9 kDa	2alg (Peach)	0.15 mM; 25 mM phosphate, 150 mM NaCl; pH 7.0	
Cupin superfamily							
Vicillin-type (7/8S globulin)	Seed storage	Cor a 11	Hazelnut*Corylus avellana*	48 kDa	2ea7 Chain A (Adzuki Bean)	0.04 mM; 50 mM phosphate, 150 mM NaCl; pH 7.5	folded
		Ara h 1	Peanut*Arachis hypogaea*	62 kDa	3fz3 (Almond)	0.03 mM; 50 mM MOPS, 20 mM NaCl pH 7.8	predominantely folded
Legumin-type (11S)	Seed storage	Cor a 9	Hazelnut*Corylus avellana*	60 kDa	3fz3 (Almond)	0.03 mM; 50 mM phosphate, 150 mM NaCl; pH 7.5	predominantely folded
		Ara h 3/4Ara h 3.0101Ara h 3.0201	Peanut*Arachis hypogaea*	60 kDa	3c3v (partial structure)	0.14 mM; 50 mM phosphate, 150 mM NaCl; pH 7.5	predominantely folded
**Animal Food Allergens**							
Casein	Ca^2+^ binding	Bos d 8	Cow's milk*Bos domesticus*	19–24 kDa		0.08 mM; 40 mM phosphate; pH 7.4	non folded
			Goat's milk*Capra hircus*	19–24 kDa		0.19 mM; 40 mM phosphate; pH 7.4	non folded
Tropomyosin	Regulator of muscle contraction	Pen a 1	Shrimp*Penaeus aztecus*	33 kDa	1c1g (Pig)	0.13 mM; 20 mM MOPS, 0.5 M NaCl; pH 7.6	non folded

* Allergen designations according to the IUIS Allergen nomenclature committee (www.allergen.org).

** Solved and available structures are listed, with their PDB entries. If models were generated then the respective template structure is given.

### Nuclear magnetic resonance spectroscopy

Allergen solutions were prepared in H_2_O/D_2_O 9∶1, (0.50 ml, with concentration ranging from 0.39 mM to 0.03 mM) in aqueous buffer systems (pH ranging from 7.0 to 7.8) as detailed in the legends for figures. The solutions were transferred into high-quality NMR tube with argon as headspace gas. Before and after the NMR experiments, the allergens were stored at 253 K (−20°C). Two High Resolution NMR experiments were carried out, using an Avance 700 spectrometer by Bruker, operating at a proton resonance frequency of 700 MHz (11.7 Tesla). The two experiments differed in the method of managing the water signal. The pulse program of the first experiment included a 1D sequence with pre-saturation to minimize the water signal [25]. The second experiment is based on gradient-based water suppression by 1D excitation sculpting using 180° water-selective pulses [25]. It increases the overall sensitivity and decreases the distortion of the baseline, but some signal near the (suppressed) water peak can be suppressed. This can be checked by the pre-saturation experiment.

For each sample and for each experiment 256 scans at 298 K (25°C) were routinely programmed, due to the low sample concentration. More scans were required for proteins with higher molecular mass and more diluted protein solutions (Mal d 3, Ara h 3/4, Cor a 9, Ara h 1, Cor a 11) as specified in the legends of the corresponding figures, where variations in the number of scans and of the field strength are reported, with the molecular weights and the molarities of the samples and the buffer conditions. The spectra were initially evaluated considering the dispersion of the signals in the regions of the methyl protons (−0.5–1.5 ppm), alpha-protons (3.5–6.0 ppm), and amide protons (6.0–10.0 ppm).

The consistency of the allergen spectra with information available in the scientific literature and with structures either deposited into the Protein Data Bank (PDB) or supported by a publication, was routinely checked. If no experimental structure was available, the comparison with a modelled structure was performed [26]–[29] and its consistency with the NMR spectra checked.

### Study of thermal stability

The stability of the nsLTPs (Mal d 3, Pru p 3 and Cor a 8) to thermal stress was studied using a 600 MHz spectrometer equipped with a probe suited to work at high temperature. A series of experiments was performed consisting of stepwise heating of each sample from 298 K (25°C) to a maximum 358 K (85°C), by 5 K intervals. After each step an NMR water pre-saturation (zgpr) experiment was launched and the spectrum evaluated. If significant structural changes were detectable, the sample was cooled to 298 K and scanned to verify whether the detected structural change was reversible. If the maximum temperature allowed by the probe had not been already reached, the sample was heated to the next threshold.

A minimum of 128 scans was programmed for each NMR experiment, extended to 512 for the most diluted samples (Mal d 3). A minimum of 20 minutes was required to reach the thermal equilibrium after each 5 K heating step. A minimum of 90 minutes was required after each cooling step and after each following heating step. All the experiments were conducted under near-neutral pH condition. The thermal stability of Pru p 3 was also studied in acidic solution (adjusting to pH 3 by addition of HCl).The pH was checked before and after the experiments.

### Modelling of allergens

The modelled structures were obtained using the fully automated protein structure homology-modelling server SWISS-MODEL [25], [27], [28] and were selected based on the protein family of the template, the sequence identity between the target and the template, the modelled residue range of the target, the QMEAN Z-Score and the QMEAN4 global score of the model [26], [30]–[32]. Models of low quality are expected to have strongly negative QMEAN Z-scores. The QMEAN describes the likelihood that a given model is of comparable quality to experimental structures. The QMEAN4 ranges between 0 and 1 with higher values indicating better models among alternative models of the same target.

## Results and Discussion

### NMR analysis

Since large chemical shift dispersions, especially in the methyl group region between 1.0 ppm and −1.0 ppm and in the amide region down-field of 8.5 ppm can be used as a reliable indicator for protein folding [10], [33] this approach was taken to assessing the structural attributes of the selected allergens. Few broad peaks around 8.3 ppm are a good indicator of a protein without an ordered tertiary structure, because this is a region characteristic of backbone amides in random-coil configuration. By contrast, several peaks, possibly narrow and resolved, dispersed beyond 8.5 ppm (8.5–11 ppm) are the signature of a rigid and ordered tertiary structure. However, the signals of the amide protons can be broadened due to chemical exchange especially at non-acidic pH. This can be a drawback at protein concentrations close to the detection limit when amide signals are to be observed. Isolated methyl group signals upfield of 0.5 ppm can be more sensitive and reliable probes for folded protein as they are not affected by chemical exchange broadening. In addition, their signal intensities are higher because three equivalent protons contribute rather than one proton of the amide group. Therefore, a wide signal dispersion in the aliphatic region of the spectrum between 1.0 and −1.0 ppm, versus a steep flank of the dominant peak at approximately 1 ppm reliably discriminates rigid and ordered tertiary structures from random-coil or flexible structures. This is because the loss of high field methyl peaks indicates a reduction in tertiary side chain organization, which can be considered complete when the NMR spectrum is characterized by few, rather narrow, but poorly dispersed peaks that are indicative of extremely mobile residues. This condition can be usually achieved as a result of protein unfolding in strongly acidic condition usually in the presence of a high concentration of urea [34].

A thermodynamically distinct state of proteins showing properties of both native and denatured proteins is “molten globule” [35]. Molten globules are considered as intermediates on the protein folding/unfolding process but in some cases, molten globules exist as stable conformers, with rigid but different tertiary structure [9]. The NMR spectrum of a molten globule protein is characterized by few, extremely broad peaks, especially for most of the backbone amide groups.

Whilst spectral signals from certain allergens (Ara h 1, Cor a 9, Cor a 11, Mal d 3) were weak and spectra were dominated by buffer signals this did not affect the readability of the critical spectral regions.

All the spectra were consistent with the corresponding structures deposited in the PDB, or with structures that could be modelled starting from the sequence and anticipated on the basis of the proteins family membership. Analysis of the 1D ^1^H-NMR spectra then allowed the classification of the allergens under study as follows.

### Prolamin superfamily - nsLTPs

**Figure 1:**
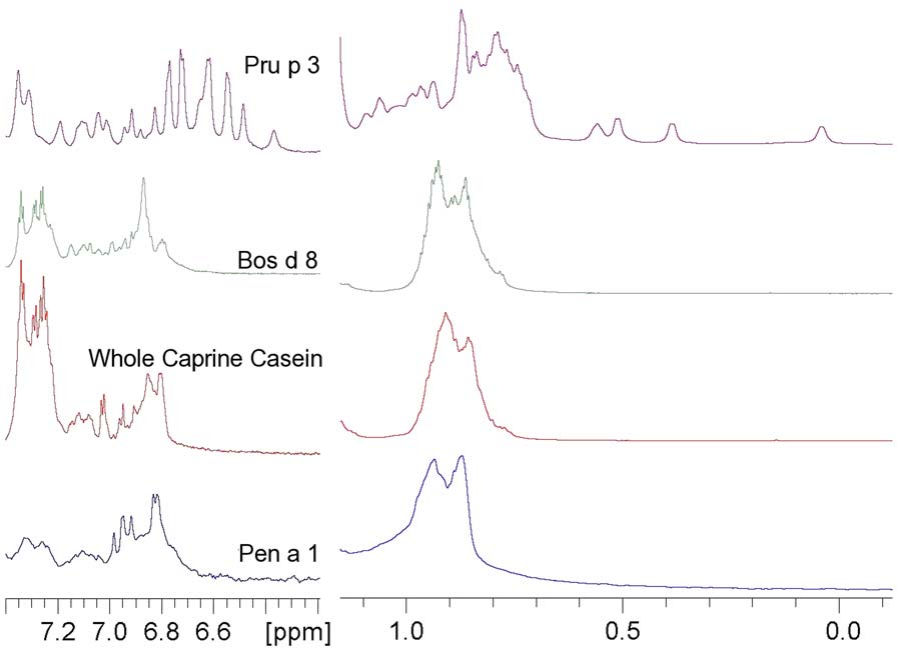
NMR spectra typical for allergens with random coil structure, compared with a spectrum of an allergen with rigid tertiary structure. Two enlarged regions of the spectra (gradient-based water suppression NMR experiment) of purified casein fraction from cow (Bos d 8, 0.08 mM, 900 MHz cryoprobe, 40 mM phosphate, pH 7.4), and goat (whole caprine casein, WCC, 0.19 mM; 40 mM phosphate pH 7.4), and of the recombinant tropomyosin from shrimp (Pen a 1, 0.13 mM, 36.4 kDa, MOPS 20 mM, NaCl 0.5 M, pH 7.6) are shown. The 0.3–1.2 ppm region includes the extreme methyl signals while the 6.2–7.4 ppm region includes the resonances of aromatic ring protons, side chain HN, and part of backbone HN; These spectra are typical of random coil structures, as indicated by the absence of signal upfield 6.6 ppm and 0.7 ppm. The same enlarged regions of the spectrum of Pru p 3 (0.39 mM, 91 AA, 9.2 kDa) are shown (top) to underline the difference between spectra of folded and unfolded proteins.

**Figure 2:**
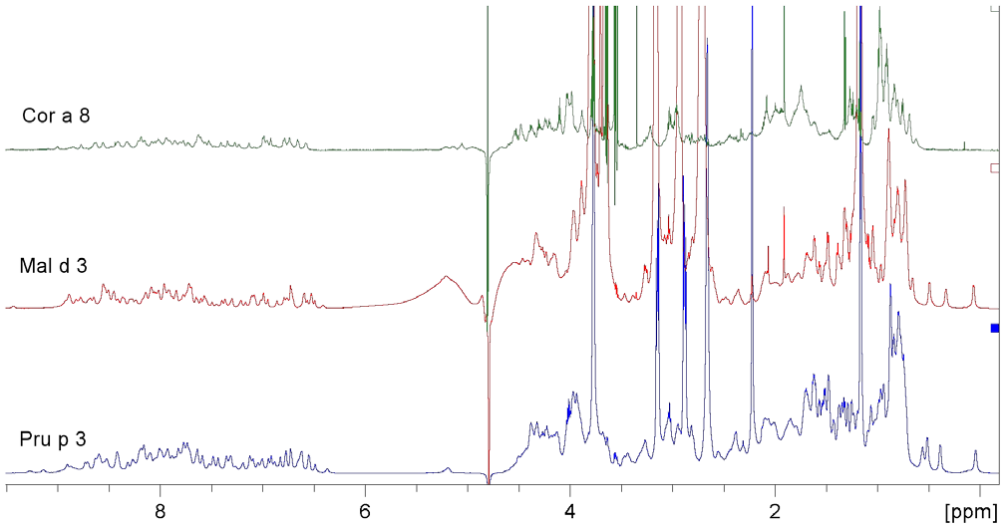
NMR Spectra of non specific lipid transfer protein, nsLTP, from peach (Pru p 3), apple (Mal d 3) and hazelnut (Cor a 8). The spectra (gradient-based water suppression NMR experiment) of nsLTPs from different sources are compared: Cor a 8 from hazelnut (115 AA, 11.8 kDa, 0.15 mM; 25 mM phosphate, NaCl 150 mM, pH 7.0), Mal d 3 from apple (115 AA, 11.4 kDa, 0.08 mM, 6504 scans; 20 mM phosphate, pH 7.4), Pru p 3 from peach (91 AA, 9.2 kDa, 0.39 mM; 25 mM phosphate, pH 7.0). These spectra indicate the presence of extended, rigid and ordered tertiary structure for all three molecules and display a high degree of similarity.

**Figure 3:**
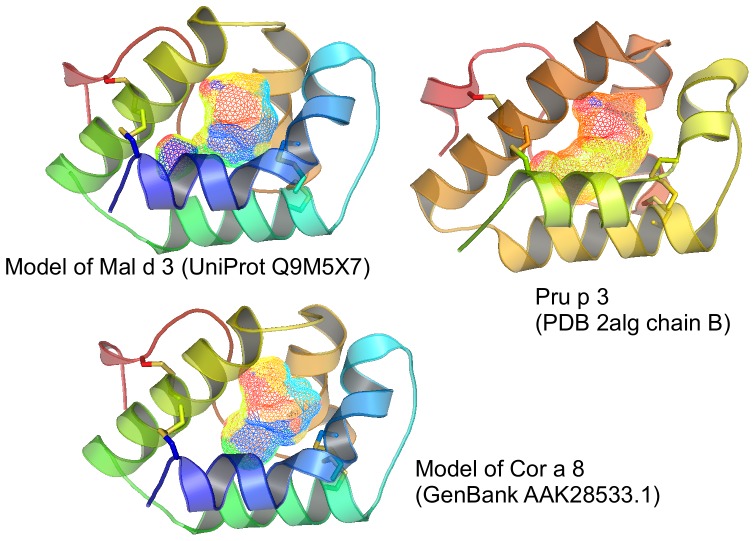
Modelled and experimental structures of nsLTPs from apple, peach and hazelnut. Models of Mal d 3 and Cor a 8 based on the experimental structure of Pru p 3 as template (PDB 2alg, chain B, 92 residues). Disulphide bonds are rendered as sticks and the internal hydrophobic cavity is rendered as a mesh surface. The modelled residue range is 25–115 for both models. QMEAN Z-score = −1.895 (Mal d 3) and −1.639 (Cor a 8).

The NMR spectra of these nsLTPs (Fig. 1 and Fig. 2) show the unmistakable features of rigid and extended tertiary structures which is not affected by low pH environment and is in line with the 2D studies performed with Pru p 3 by Wijeshina-Bettoni et al [14]. There are many narrow and resolved peaks throughout the whole spectral window, notably in the methyl and amide-aromatic regions. The spectra of these allergens are very similar: for example, the extreme methyl region between 1 and 0 ppm and the aromatic region between 6 and 8 ppm. This fact gives experimental support to the similarity of the modelled structures of Mal d 3 and Cor a 8, and the experimental structure of Pru p 3 (Fig. 3), as expected from the high degree of sequence homology and conserved cysteine residues forming disulfide bridges. Thus Mal d 3 (Uni Prot entry: Q9M5X7), and Cor a 8 (Uni Prot entry: Q9ATH2), share 81.3% and 59% sequence identity with Pru p 3 (Uni Prot entry: P81402), respectively. The overall intact 3D structure is relevant for IgE-binding activity of Pru p 3 and is reduced when alkylation and reduction of the protein was performed [36]. Therefore, Pru p 3 was subjected to stepwise heat treatment to investigate whether heating processes have an impact on its overall structure.

**Figure 4:**
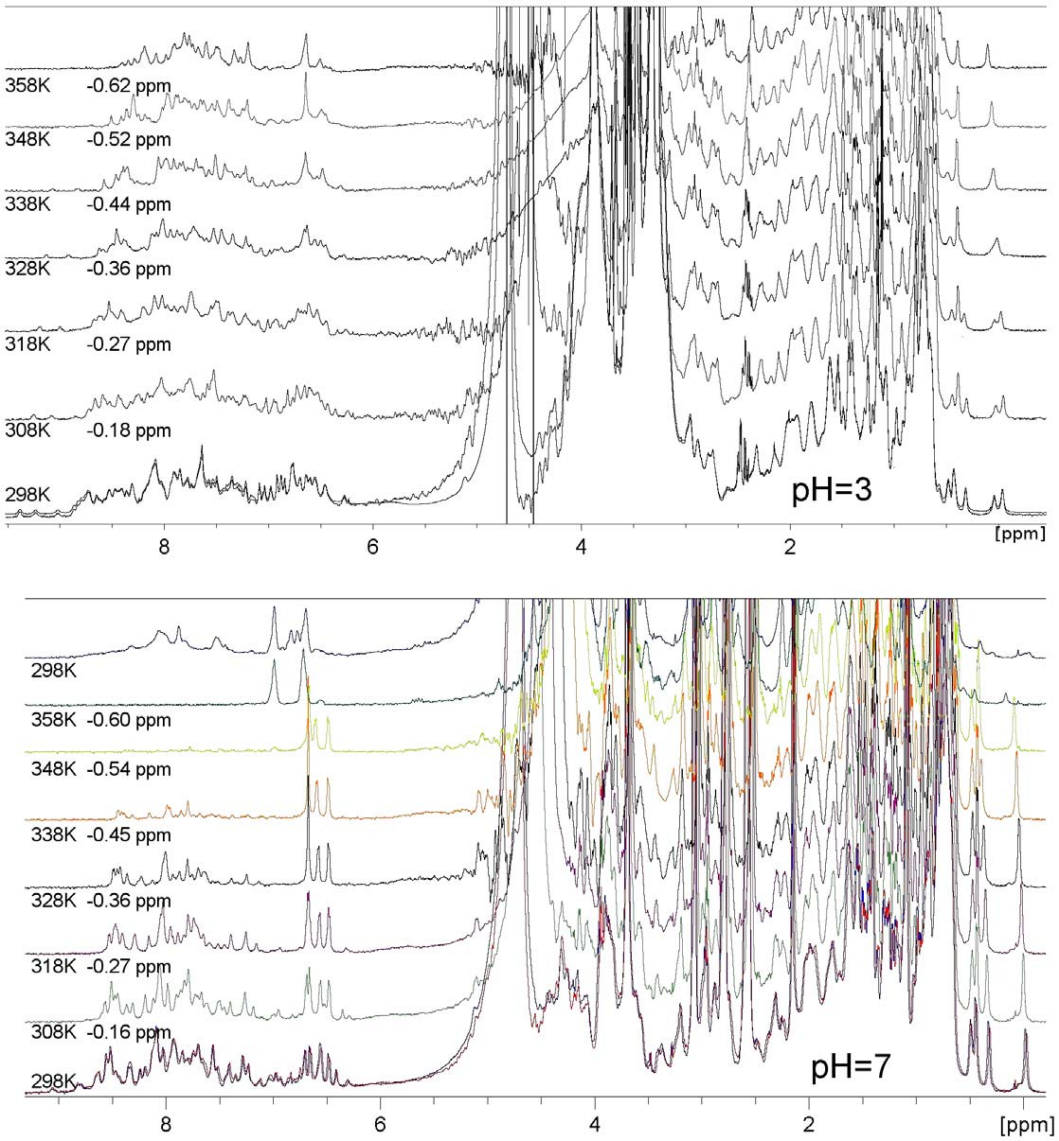
NMR spectra of nsLTP from peach, Pru p 3 under thermal treatment in acidic and near-neutral pH conditions. Heating of Pru p 3 (0.39 mM, 91 AA, 9.2 kDa) under acidic (pH 3.0) and neutral (pH 7.0) conditions; pre-saturation NMR experiments. To improve readability, the spectra at T>298 K are aligned with the reference spectrum at 298 K (25°C), by shifting them upfield as indicated after the temperature. pH 3.0 bottom: NMR spectra of Pru p 3 scanned at 298 K before and after heating up to 358 K (85°C). pH 7.0, bottom: NMR spectra of Pru p 3 scanned at 298 K before and after heating up to 358 K. top: spectrum scanned at 298 K after 358 K heating. In neutral conditions the protein undergoes irreversible denaturation, in the 80°C–85°C range of temperature.

During thermal treatment the spectra gradually change towards the denatured state. Under acidic conditions (Fig. 4, pH 3) the increasing loss of tertiary structure of Pru p 3 is apparent from the decreasing dispersion of peaks either in the methyl region (around 0 ppm) or in the amide-aromatic region (6–10 ppm). Irreversible denaturation is not detectable until 358 K (85°C) and the 298 K (25°C) spectra, scanned before and after heating, overlap perfectly. These data are in good agreement with the studies on heat resistance of barley LPT from Perrocheau et al. [37]. Under neutral conditions (Fig. 4, pH 7) two major differences can be observed during thermal denaturation of natural Pru p 3. The former is an irreversible structural transition occurring between 353 K (80°C) and 358 K (85°C): the final room temperature spectrum shows only a few broad and poorly dispersed peaks in the amide-aromatic and in the methyl region and the absence of resonances upfield and downfield. The latter represents the disappearance of amide resonances (7–9 ppm) as the temperature rises. This is due to proton exchange with the solvent, and depends on pH, temperature and accessibility of exchangeable protons, which increases with unfolding, and can be due to the transition to a molten-globule state. Native Mal d 3 shares the same behaviour as Pru p 3 when heated in neutral conditions (data not shown). Heating was stopped at 348 K (75°C) for Cor a 8 due to instrumental problems. The protein refolds to the 25°C tertiary structure after the heat-induced unfolding at the maximum tested temperature (data not shown).

### Cupins – seed storage globulins

**Figure 5:**
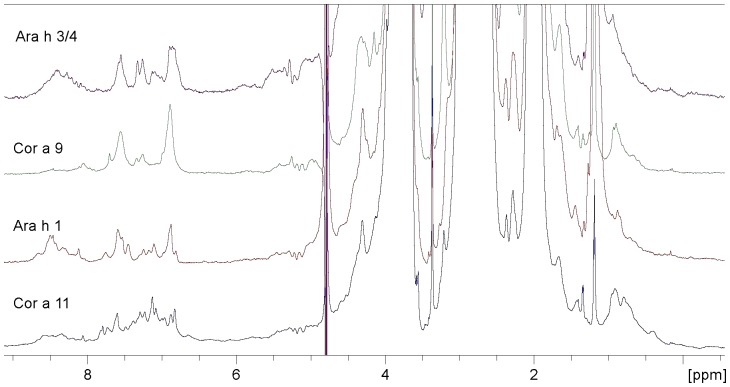
NMR Spectra of globulins from peanut and hazelnut. The spectra (gradient-based water suppression NMR experiment, 848 scans) of selected globulins from different sources are presented: Ara h 3/4 (mixture of isoforms of 11S globulins from peanut, 37.0 kDa, putative concentration 0.14 mM; 50 mM phosphate, NaCl 150 mM, pH 7.5), Cor a 9 (11S globulin from hazelnut, 40 kDa, 0.03 mM, 50 mM phosphate, NaCl 150 mM, pH 7.5), Ara h 1 (7/8 S globulin from peanut, 63.5 kDa, 0.03 mM, 50 mM MOPS, NaCl 200 mM, pH 7.8), Cor a 11 (7/8S globulin from hazelnut 48 kDa, 0.04 mM, phosphate 50 mM, NaCl 150 mM, pH 7.5), Signals, though basically unresolved due to high MW and possible aggregation, can be observed in the extreme methyl region of all the spectra (around 0.5 ppm) which indicate of regions of rigid tertiary structure. The presence of extended disordered regions must also be considered, due to the few broad peaks that can be observed in the whole spectral window, especially in the HN region between 6 and 8 ppm and especially for Cor a 9. On the contrary the same region of Cor a 11 is populated by several more resolved and narrow resonances, indicative of rigid and ordered regions. Both, Cor a 9 and Cor a 11 show a noticeable signal broadening in the backbone NH region (8.0–8.7 ppm), indicating the presence of the molten globule state in solution. In the Ara 3/4 spectrum, narrow, weak signals, can be observed on an envelope of broader peaks. They could be due to the lighter (non-aggregated) component of the mixture.

The higher molecular mass of globulins as compared to nsLTPs and their propensity to aggregate into more complex quaternary structures seriously limits the resolution of their NMR spectra (Fig. 5). This could be a particular problem for Cor a 9, due to the possible presence of the hexameric form. A series of signals, though basically unresolved, can be observed in the extreme methyl region of all the spectra (around 0.5 ppm) indicating regions of rigid tertiary structure. On the other hand, the presence of extended mobile regions must be considered, due to the few broad peaks that can be observed in the whole spectral window, notably in the HN region between 6 and 8 ppm. By contrast, the same region of the Cor a 11 spectrum is populated by several better resolved and narrow resonances that give a clear indication of rigid and ordered regions. Both, Cor a 9 and Cor a 11 show a noticeable signal broadening in the backbone NH region between 8.0 and 8.7 ppm, that could indicate the presence of a molten globule state. Narrow, weak signals are observed against a background of broad peaks in the Ara h 3/4 spectrum which could be due to the presence of both lighter monomers and more massive aggregations in the mixture (Fig. 5).

**Figure 6:**
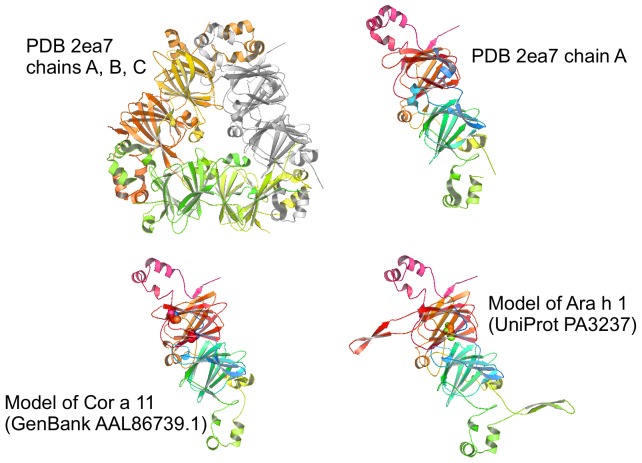
Modelled and experimental structures of 7/8S globulins from peanut, hazelnut and adzuki bean. Models of 7/8S globulins from peanut (Ara h 1, 614 residues) and hazelnut (Cor a 11, 448 residues), both based on 7S globulin from adzuki bean (PDB 2ea7 chain A) as template. Either the monomer (chain A) or the trimer (chain A rendered with the same orientation, in light gray) of the template are shown. The presence of mobile parts is indicated by the many extended loops of the models, corresponding to similar extended loops or to unresolved regions of the template. Modelled residue range: 161–583 (Ara h 1), 55–435 (Cor a 11). Sequence Identity [%]: 49 (Ara h 1), 32 (Cor a 11). QMEAN Z-score = −1.614 (Ara h 1), −0.956 (Cor a 11). QMEAN Z-score of Ara h 1 = 0.669 (0.600 if it is modelled on PDB 2cv6 chain A, corresponding to 8S globulins of mung bean).

Up to now two sequences of 7/8S globulin Ara h 1, from peanut (63.5 kDa) (UniProt entries P43237 and P43238) were characterised [38]. Very recently, the structure from the Ara h 1 core region without the N-terminal extension and the C-terminal flexible region was determined [39]. Both isoforms could be modelled using the 7S globulin from adzuki bean [40] as a template (PDB accession 2ea7), with 48% of sequence identity (Fig. 6). The template and the modelled monomer can be divided into two similar modules (N- and C-terminal), each showing an extended loop domain. The models could be verified by the crystal based analysis of the Ara h 1 core fragment [39], [41]. Already identified IgE epitopes, were mapped on the models and it became evident that some of these epitopes were only surface exposed when the trimer formation was disrupted [42].

**Figure 7:**
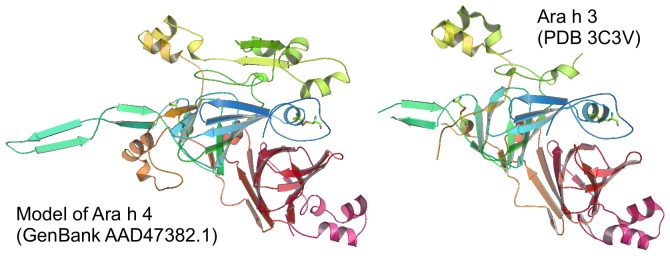
Modelled and experimental structures of Ara h 3/4 isoforms. Experimental structure of Ara h 3/4 (PDB 3c3b) and model of its sequenced isoform (Q9SQH7 GenBank AAD47382.1; 530 residues) based on PDB 3c3v as template. The presence of mobile parts is indicated by the many extended loops of the model, corresponding to similar extended loops or to unresolved regions of the template. Disulphide bonds are rendered as sticks. Modelled residue range: 21–521. Sequence Identity [%]: 75. QMEAN Z-score = −1.933.

A partially resolved structure (PDB entry 3c3v) has been reported as Ara h 3 (now designated: Ara h 3.0101; Swiss prot Entry: O82580) [43]. It consists of an N-terminal domain and a C-terminal domain. Three regions of the N-terminal domain and the tail of C-terminal domain are disordered (Fig. 7). Using this structure as a template a model was built of the isoform reported as Ara h 4 (now designated: Ara h 3.0201; Swiss Prot entry: Q9SQH7) [44]. The model which shares 75.5% sequence identity with “Ara h 3” (Fig. 7) was calculated including the unresolved regions of the experimental structure, and is consistent with the presence of extended, partially structured, mobile regions.

IgE epitopes were identified on the surface of Ara h 3, some of them specific for the peanut allergen and some of them shared with other legumin type allergens highlighting the molecular basis for cross-reactivity [45].

**Figure 8:**
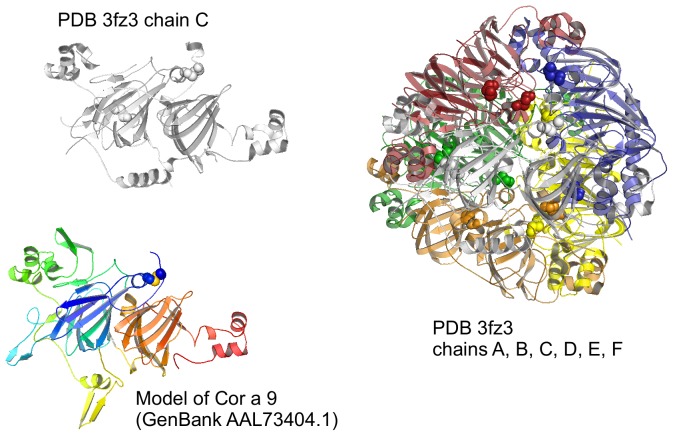
Modelled and experimental structures of 11S globulins from hazelnut and almond. Model of Cor a 9 (11S globulin from hazelnut), based on template PDB 3fz3 (11S globulin from almond) chain C (515 residues). Disulphide bonds are rendered as spheres. Both the monomer and the hexamer of the template are shown. The template's chain C is rendered in light gray, with the same orientation, either as monomer or in the hexamer. The presence of mobile parts is indicated by the many extended loops of the model, corresponding to similar extended loops or to unresolved regions of the template. Modelled residue range: 34 to 496. Sequence Identity [%]: 51. QMEAN Z-score = −3.084.

No experimental structure of hazelnut globulins is known to date. Again a homology model was built (Fig. 6) using the Cor a 11 sequence [46] starting from its 48 kDa precursor sequence (Swiss Prot entry: Q8S4P9), with 32.5% of identity with the template PDB 2ea7, chain A, corresponding to the adzuki bean 7S globulin [40]. The model is trimeric, with each monomer formed by two very similar modules and each module containing an extended loop domain. Similarly a model was built using the Cor a 9 sequence (GenBank: AAL73404.1) which has 51.8% sequence identity to the almond globulin [47], [48] (PDB 3fz3) (Fig. 8). The structure of this template, and of the derived model, shows similarities with the structure of the 11S globulin Ara h 3/4 (Ara h 3.0101 and Ara h 3.0201; Fig. 7 and Fig. 8).

Narrow, weak signals in are observed against a background of broad peaks in the Ara 3/4 spectrum which could be due to the presence of both lighter monomers and more massive aggregations in the mixture (Fig. 5).

The spectra of all of the globulins provide experimental support for their modelled structures and are consistent with our knowledge of the structural characteristics of this family of proteins including their tendency towards aggregation, the ubiquity of isoforms, and the presence of extended mobile regions in their tertiary structure.

### Tropomyosin and Caseins

**Figure 9:**
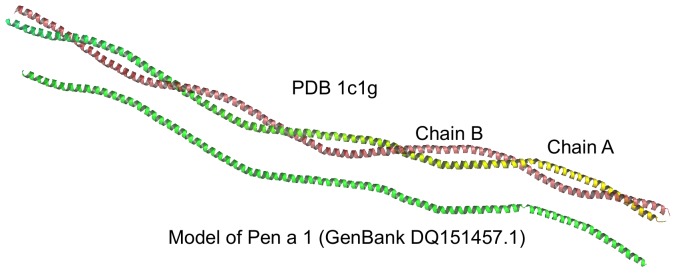
Structures of tropomyosin from brown shrimp (modelled) and pig (experimental). Model of Pen a 1, the tropomyosin from brown shrimp (284 residues) based on tropomyosin (PDB 1c1g chain A) from cardiac muscle of pig (*Sus scrofa*). Tropomyosins can pass from a coiled coil to molten-like structure before complete dissociation of the two chains. Chains are flexible and can be unfolded at physiological temperature. Modelled residue range: 1 to 283. Sequence Identity [%]: 56. QMEAN Z-score = −0.371. QMEAN score 4 = 0.755 (0.732 on tropomyosin from striated-muscle of *R. norvegicus* 2b9c chain A).

The NMR spectra of the sample of Pen a 1 under study and the spectra of caseins are very similar. These spectra (Fig. 1) show the typical characteristics of random coil structures with extended regions of the spectral window being devoid of signals and a few broad peaks at characteristic intervals. The dominant methyl peak at approximately 1 ppm is a distinctive signature of a random-coil or extremely flexible structure. Single narrow signals can be observed on the envelope of broad peaks which are not resolved to the baseline because they are confined in narrow spectral ranges, confirming the high mobility of the residues of these samples. A model was constructed of Pen a 1 (Accession No. DQ151457.1) [49] based on the structure of pig cardiac muscle tropomyosin [50] (PDB 1c1g, chain A) with which it shares 56.2% sequence identity (Fig. 9). This has a coiled-coil structure which can undergo a transition from a coiled coil to molten-like state before complete dissociation of the two chains, chains that are flexible and can be unfolded at physiological temperature [51]. This could explain the observation that the NMR spectrum is characteristic of a mobile, unfolded structure. Detailed studies on the epitope mapping of this major allergen identified linear IgE epitopes with increased antibody binding activity when grafted onto non allergenic tropomyosin [17].

The spectra of the caseins were similarly characterised by a lack of signals between 6.2 and 6.6 ppm and 0.0 and 0.7 ppm, respectively (Fig. 1) indicative of their highly mobile structure. The fact that unfractionated caseins are mixtures of four different molecules is not apparent because alpha-s1-casein, beta-casein, alpha-s2-casein and k-casein [19], all share similar molecular weight and lack secondary and tertiary structure, and therefore their residues share very similar structural environment as confirmed also by the great similarity of all these spectra.

### Structural classification of allergens

On the basis of the NMR analysis the proteins could be classified into three groups. The first group included those molecules whose spectra showed the unquestionable signature of their tertiary structure: nsLTPs (Fig. 1 and Fig. 2) and the hazelnut 7/8S globulin, Cor a 11. However, the NMR data indicate that the latter may contain disordered stretches (Fig. 5) as has been observed for seed storage globulins from other plant species such as soybean [52].

The second group included those allergens whose spectra clearly showed no fingerprints for tertiary structure (Fig. 1). These were whole caseins from cows' and goats' milk (Bos d 8, WCC) and tropomyosin from brown shrimp (Pen a 1). The spectra for caseins are consistent with other observations of protein mobility which has led to them being termed rheomorphic [19].

The third group included allergens whose spectra revealed an intermediate structure: the 11S globulin Cor a 9 and the 7/8S globulin Ara h 1. Their spectra (Fig. 5) indicated that the protein could be structured with well organized rigid regions with an ordered tertiary structure, interspersed with disordered regions (as indicated by temperature factors in the x-ray structures of certain seed storage globulins and the fact certain regions had not been resolved and appear to be highly mobile even in a crystal lattice [41]. Alternatively these data indicate the sample could be a mixture of folded and unfolded or differently folded proteins. The spectrum of the 11 S globulin Ara h 3/4 (Fig. 5) also indicated the presence of both disordered and ordered parts.

It is worth noting that some of the allergens investigated displayed molecular masses, (e.g. 60 kDa without considering possible aggregations) that can be considered beyond the limitations of the method and the instruments adopted.

### Concluding remarks

High quality allergen batches with defined physicochemical features are essential for reliable and sensitive *in vitro* diagnosis for allergy. The 3-dimensional structures of the most important allergen families have important impacts on their IgE binding activity. It has become evident that food processing can affect the structure and thus allergenicity. Therefore, methods to assess the structural integrity of purified allergens are urgently needed. The present study has shown that 1D NMR can be used to generate a fingerprint containing the overall structural and dynamic information of allergenic proteins in a high throughput manner.
